# Standardizing hyperphagia outcomes: addressing the need for rigorous causality assessment

**DOI:** 10.1097/MS9.0000000000005372

**Published:** 2026-08-12

**Authors:** Sohail Memon

**Affiliations:** College of Pharmacy, Liaquat University of Medical & Health Sciences (LUMHS), Jamshoro, Sindh, Pakistan

**Keywords:** causality assessment, hyperphagia, Naranjo algorithm, pharmacovigilance, Prader–Willi syndrome, Vykat XR

*Dear Editor*,

The recent approval of Vykat XR (diazoxide choline-extended release) for hyperphagia associated with Prader–Willi syndrome (PWS) marks a pivotal milestone^[^1^]^. While clinical trials establish basic safety baselines, translating this first-in-class therapy into real-world settings presents unique monitoring challenges that demand a deeper pharmacological critique.

PWS presents a uniquely problematic landscape for standard pharmacovigilance. This neurogenetic disorder features innate hypothalamic dysfunction, hyperphagia-driven metabolic syndrome, and a baseline phenotype that heavily overlaps with severe drug toxicities^[^2^]^. When Vykat XR an adenosine triphosphate (ATP)-sensitive potassium (K_ATP) channel opener is introduced into complex regimens involving psychotropics and growth hormones, standard polypharmacy tracking proves insufficient^[^1,2^]^. Vykat XR’s landmark clinical trials revealed notable adverse event profiles: peripheral edema occurs in 27–34% of patients (ranging from mild fluid retention to severe localized swelling), while glycemic disturbances, primarily hyperglycemia, affect roughly 17–22% of cohorts due to the drug’s intentional inhibition of pancreatic beta-cell insulin secretion^[^3,4^]^. The fundamental limitation of existing clinical trial data is its highly controlled, short-term nature; it cannot fully predict how these toxicities compound over years of multi-agent treatment or differentiate a drug-induced glycemic spike from the patient’s underlying genetic progression toward type 2 diabetes^[^1,3^]^.

Without objective causality frameworks, clinicians risk either prematurely discontinuing a highly effective drug due to syndromic misattribution or missing a cumulative drug interaction. To realistically integrate these tools into routine, fast-paced clinical practice, we propose a tiered, electronic health record–driven workflow^[^5^]^. During routine quarterly multidisciplinary PWS clinic visits, nursing or allied health staff can administer a simplified, automated five-item digital Naranjo screening checklist during initial triage when peripheral edema or shifted HbA1c values are logged^[^5,6^]^. Any score yielding a “possible” or “probable” relationship is seamlessly escalated to the clinical pharmacist or attending physician for definitive World Health Organization-Uppsala Monitoring Centre matrix adjudication^[^6,7^]^. Implementing this structured, low-burden pharmacovigilance model bridges the gap between clinical data and real-world execution, securing the long-term safety of this vulnerable population.

## Ethical approval

Ethical approval was not required for this Letter to the Editor as no human or animal subjects were directly involved.

## Consent

Not applicable.

## Data Availability

Data sharing is not applicable to this article as no datasets were generated or analyzed during the current study.
